# Developing user personas to capture intersecting dimensions of disadvantage in older patients who are marginalised: a qualitative study

**DOI:** 10.3399/BJGP.2023.0412

**Published:** 2024-03-05

**Authors:** Laiba Husain, Teresa Finlay, Arqam Husain, Joseph Wherton, Gemma Hughes, Trisha Greenhalgh

**Affiliations:** The Healthcare Improvement Studies Institute research fellow;; Nuffield Department of Primary Care Health Sciences, University of Oxford, Oxford, UK.; School of Medicine, Wayne State University, Detroit, MI, US.; Nuffield Department of Primary Care Health Sciences, University of Oxford, Oxford, UK.; School of Business, University of Leicester, Leicester, UK.; Nuffield Department of Primary Care Health Sciences, University of Oxford, Oxford, UK.

**Keywords:** aged, digital disparities, digital equity, digital healthcare, focus groups, primary care

## Abstract

**Background:**

Remote and digital services must be equitable, but some patients have difficulty using these services. Designing measures to overcome digital disparities can be challenging for practices. Personas (fictional cases) are a potentially useful tool in this regard.

**Aim:**

To develop and test a set of personas to reflect the lived experiences and challenges that older people who are disadvantaged face when navigating remote and digital primary care services.

**Design and setting:**

Qualitative study of digital disparities in NHS community health services offering video appointments.

**Method:**

Following familiarisation visits and interviews with service providers, 17 older people with multiple markers of disadvantage (limited English, health conditions, and poverty) were recruited and interviewed using narrative prompts. Data were analysed using an intersectionality lens, underpinned by sociological theory. Combining data across all participant interviews, we produced personas and refined these following focus groups involving health professionals, patients, and advocates (*n* = 12).

**Results:**

Digital services create significant challenges for older patients with limited economic, social, and linguistic resources and low digital, health, or system literacy. Four contrasting personas were produced, capturing the variety and complexity of how dimensions of disadvantage intersected and influenced identity and actions. The personas illustrate important themes including experience of racism and discrimination, disorientation, discontinuity, limited presence, weak relationships, loss of agency, and mistrust of services and providers.

**Conclusion:**

Personas can illuminate the multiple and intersecting dimensions of disadvantage in patient populations who are marginalised and may prove useful when designing or redesigning digital primary care services. Adopting an intersectional lens may help practices address digital disparities.

## Introduction

The shift to remote and digital forms of health care is dramatically changing the landscape of UK primary care. This requires practices to find ways of ensuring that nobody is disadvantaged. But older people in particular can have problems accessing such services, especially when they are disadvantaged and have complex needs.^1^^–^6

A recent literature review by our team found multiple studies that showed that certain subgroups such as older, poor, minority ethnic groups and those with disabilities were less able to access remote and digital health services.^7^^–^11 Older age, low socioeconomic status, ethnic minority status, and low digital or health literacy all contributed additively to lower rates of telehealth use,^8^^,^^12^^–^15 findings that align with the literature on the cumulative effect of social determinants (for example, poverty, poor housing, lack of educational opportunity, racism, and discrimination) on health generally.^16^^–^20 Some scholars have proposed that access to broadband should be considered a new social determinant of health as so many services now require digital access.^21^ Some studies have proposed lists of ‘barriers’ (for example, device and skill shortages) and ‘facilitators’ (for example, skill training) to inform approaches to enhancing access for disadvantaged groups;^22^ however, such recommendations may overlook the complexity and pervasiveness of structural disadvantage. Indeed, there is some evidence that well-intentioned approaches built on a ‘deficit’ model (that is, one that assumes that digital disparities can be rectified through specific interventions aimed at individuals’ lack of devices, connectivity, skills, and confidence, for example) may inadvertently worsen digital disparities.^23^

A more fruitful approach, which reflects the theoretical literature on intersectionality (the notion that multiple markers of disadvantage combine in any individual to give a singular and unique lived experience),^24^ is to undertake detailed qualitative research into how people experience disadvantage and then design (and, preferably, codesign) services that take account of that experience. Our review showed that few such studies yet exist.^9^ There is thus an important gap in the literature to explore the lived experience of digital disparities from the perspective of the patient who is disadvantaged.

**Table:** How this fits in

Equity is an important core value in primary care, but meeting the needs of patients who are multiply disadvantaged is increasingly difficult as services become more digitised. User personas (fictional cases based on empirical data that draw together and illustrate the multiple intersecting elements of disadvantage) could help practices better plan for the needs of disadvantaged groups.

User personas are fictional but representative profiles that encapsulate different segments of a target population.^25^ They serve as design tools or guiding archetypes that providers can use to develop a more nuanced understanding of a group’s needs, improve the delivery and coordination of care, and help ensure culturally competent, patient-centred care.^26^ They have been widely used in digital exclusion work beyond health care^27^^,^^28^ and in health care to map user journeys,^29^^–^32 but there is little published literature on their use by healthcare providers to understand and address digital health disparities in marginalised groups. This study aimed to address this gap.

Our research questions were:

1. how do older people with multiple markers of disadvantage experience remote and digital health care? And
2. can these varied experiences be synthesised in user personas in a way that informs the design of more equitable services?

## Method

### Governance

This work is part of a PhD by the first author, funded by The Healthcare Improvement Studies Institute and linked to a wider programme of work on remote health care.^33^

**Figure 1.**
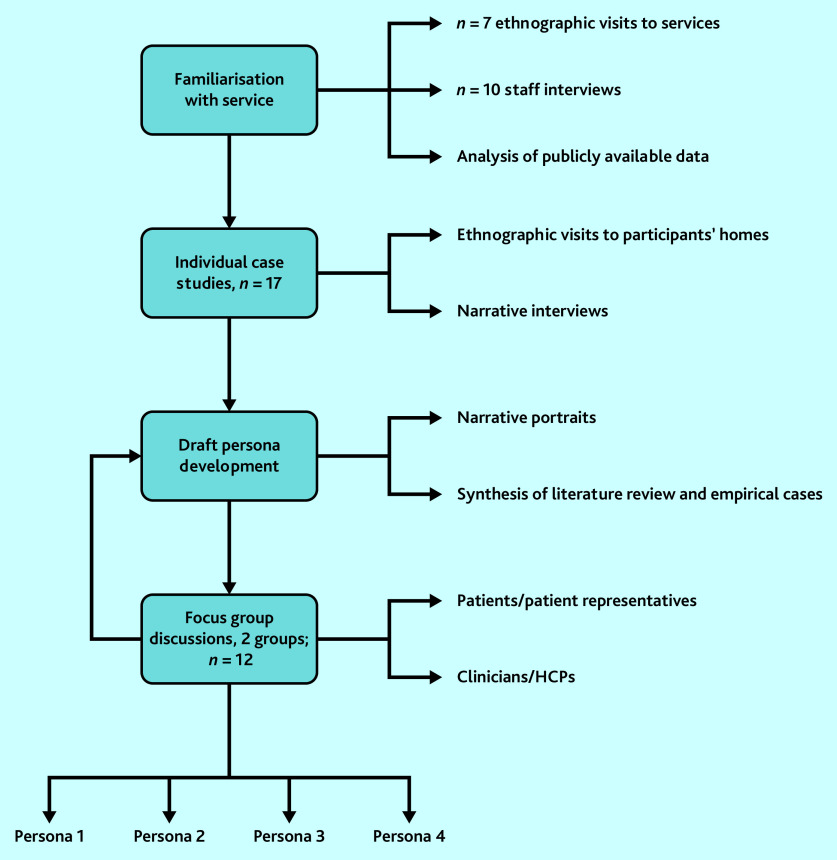
Study flowchart. HCP = healthcare professional.

The methods are summarised in Figure 1 and described below.

### Study site and familiarisation

Familiarisation visits were conducted across seven services in North East London and encompassed staff interviews and analysis of publicly available data to evaluate their suitability as study sites. This evaluation process checked for key criteria including the service’s engagement with remote consultations, location in a low-deprivation area, patient population diversity, and high proportion of older residents. This process led to the study site selection of a respiratory service in Redbridge who were trialling a pulmonary rehabilitation programme that included virtual consultations over Zoom. This meant that, unusually, video consultations were being offered to patients who were mostly older, first-generation immigrants and of low income, as well as chronically unwell. Those who could take part were loaned a device with internet access. Through staff at this service, other services (clinical health psychology) and community organisations (Age UK and Healthwatch) joined the study and supported recruitment of participants.

### Sample selection and recruitment

In total, 17 participants were selected using purposive sampling based on the following inclusion criteria: aged ≥65 years, living in a locality with an Index of Multiple Deprivation score of 1–2 (indicating they live in localities facing the highest levels of socioeconomic deprivation), had chronic health condition(s), and spoke limited English but were able to converse in a language spoken by the researcher (Hindi, Bangla, or Urdu). Potential participants were first approached by a staff member known to them and invited to consider the study. Those who showed interest were telephoned by the researcher who explained the study in their preferred language before seeking informed consent to participate.

### Data collection

Participants were visited at home. In-depth narrative interviews were conducted in the participant’s preferred language and lasted 60–120 min. The format was narrative — participants were asked to tell their story in chronological order, using open-ended questions to elicit in-depth responses. Topics covered included their health conditions and experiences, accessing healthcare services and technology, and remote consultations. Many participants also chose to talk about their families, culture, and experiences of immigration. Contemporaneous field notes were made.

### Theoretical approach

We drew primarily on Crenshaw’s theory of intersectionality which holds that systems of oppression are inherently bound together thus creating singular social experiences for people who bear the force of multiple systems.^24^ In relation to health disparities, for example, intersectionality relates to *‘intersections of individuals’ multiple identities within social systems of power that compound and exacerbate experiences of ill health’*,^34^ thus recognising that health is shaped by a multidimensional overlapping of factors such as race, class, income, education, age, ability, sexual orientation, immigration status, ethnicity, indigeneity, and geography. An individual’s experiences are understood in relation to their identity (the multiple ways they present themselves to others, such as being Muslim), positionality (the multiple social positions they occupy, such as being a wife and/or mother), and oppression (the multiple socio-structural oppressions they encounter, such as discrimination).

### Data analysis and persona construction

Interviews, often conducted in both English and the participant’s native language, were translated and transcribed verbatim by the first author. Any non-English segments were first translated into English, whereas ‘broken English’ was transcribed as-is, if the meaning was clear. To maintain narrative sequence, the transcripts were minimally restructured, ensuring a coherent storyline.

The collected data were organised using NVivo (Version 1.6.2) and analysed thematically with attention to narrative (life story) and feminist (for example, societal power structures) perspectives. This involved identifying patterns and themes in the participants’ responses and coding the data into categories. For example, the quote *‘I don’t know how any of this works. Aamir takes care of all this booking my appointments, he does it online, I don’t know how to do any of it. It’s very confusing. I get scared to do it myself’* was initially coded as: relying on family member for digital tasks (code 1) and lack of confidence with technology (code 2). These codes were grouped into the category ‘loss of autonomy’. From an intersectional lens, this category reflects diminished agency because of facing multiple axes of disadvantage (for example, poverty, low system literacy, and cultural gender roles). The thematic data were then reconstructed into narrative portraits that interwove the key experiences, challenges, and perspectives described by participants. This approach was used to preserve the focus on the individual both in analysis and in how findings were reported. To that end, the first author explored different and creative ways of summarising, representing, and reconstructing participant narratives (for example, pictorially as personas and as written stories).

In constructing the personas, the underlying themes were carefully analysed and integrated, ensuring that they accurately reflect the lived experiences and challenges of the individuals represented. For the scope of this study (the central focus of which is the persona methodology), we opted to present selected qualitative findings mainly in the form of narrative personas. More detailed qualitative findings will be presented in a separate study. The persona development served dual purposes: as an analytical approach to understand and interpret the interacting themes and intersections, and as a means of communicating and discussing these themes with users and stakeholders.

### Focus groups

Two focus group refinement sessions were conducted with a total of 12 healthcare professionals, patients, and patient representatives. The first author facilitated the groups, introducing the personas alongside a narrative of their personal and health history, and invited feedback and insights. Participants were encouraged to ask questions about the personas and the discussion flowed naturally from the questions with minimal interference from the facilitator. Both focus groups lasted for almost 2 h. An important goal of these sessions was to ensure that provider and advocacy perspectives were incorporated into the development of the personas, increasing the probability that stakeholders would find them useful.

## Results

The 17 participants were aged between 65 and 87 years; all were first-generation immigrants from South Asian countries (Pakistan, Bangladesh, Nepal, Afghanistan, and India). They lived in modest accommodation (Index of Multiple Deprivation decile range 1–2), typically a rented flat with a multigeneration family. All explained that they had difficulty accessing services and struggled in various ways with remote encounters and video consultations in particular, even though they had been loaned a digital device. The ethnographic home visits further revealed meaningful context about the participants’ realities including cramped living conditions, tangible markers of cultural heritage, and dynamics of multigenerational cohabitation.

**Figure 2.**
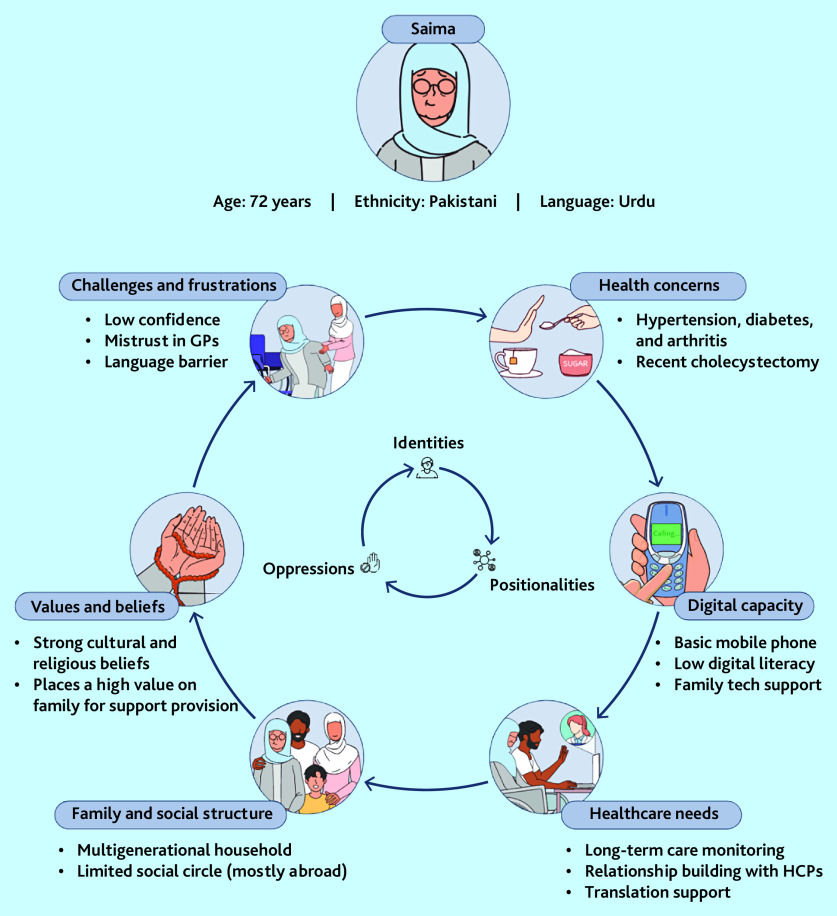
Saima user persona. HCP = healthcare professional.

We generated four user personas to capture the variety of identities, positionalities, and oppressions in our sample. Box 1 shows the narrative portrait of one of these, Saima, a (fictitious) user persona. Figure 2 shows the same persona in the pictorial form we used to prompt discussions in the focus groups.

**Box 1.** Narrative portrait of user persona Saima

Saima is 72 years of age; she has lived in the UK for 40 years. She and her husband moved from Pakistan with their two young boys in 1983 in search of a better job for him. She sees herself simultaneously as an older person, a mother, a wife, and a Pakistani Muslim woman. Although Saima was reluctant to move, she put aside her feelings but never fully adjusted to life in the UK. As her children grew up and moved away Saima felt a significant loss of purpose in her life. Her mental health and (subsequently) her physical health declined and she developed hypertension, arthritis, diabetes, and an inflamed gallbladder:
*‘I wouldn’t call this place* [UK] *home. As a mother and wife, I did what was best for my family and moved at the time. But now the children have grown up, my days are empty and I am always sick here. It’s all catching up to me in my old age.’*
Following her husband’s death, Saima moved in with her eldest son and his family. This transition brought both relief (easing of financial pressure) and a sense of cultural fulfilment, as there was an expectation for eldest sons to assume responsibility for their ageing parents. However, it also had an unintended consequence: she became reliant on her son, Aamir, for all her needs. Saima found herself becoming a passive participant in her own healthcare journey, with Aamir taking charge of booking appointments, speaking to healthcare providers, and making decisions on her behalf. With the onset of the COVID-19 pandemic, Aamir quickly adapted to digital ways of working (e-consults, telephone consultations, and video appointments) to ensure his mother’s healthcare needs were met efficiently. But Saima felt overwhelmed and disconnected from these technological modes of access. She had always relied on visits to her familiar family doctor and liked to take traditional herbal remedies. The sudden shift to a technologically driven healthcare system made her feel even more detached from her own treatment plan:
*‘I don’t know how any of this works. Aamir takes care of all this booking my appointments, he does it online, I don’t know how to do any of it. It’s very confusing. I get scared to do it myself.’*
With Aamir by her side, Saima did have a video consultation with her GP a few months ago, but it left her feeling disillusioned. As she was used to seeing her familiar GPs in her local practice before the pandemic, she felt unnerved seeing a strange floating face on her son’s laptop asking about her arthritic knee. The Wi-Fi connection was unstable, causing lapses in communication and she did not recall any small talk or pleasantries exchanged. The exchange left Saima feeling upset and disconnected:
*‘He* [the GP] *didn’t even look at me. No one tells me what’s happening. The GP talks fast, I cannot understand … they are not interested in discussing with me — after all, who am I?* [laughs].*’*
Saima’s long-standing reliance on a known GP, alongside her limited digital literacy, preference for traditional remedies, and cultural taboos surrounding mental health, combine to present substantial obstacles for her in adapting to a technology-driven service with an unfamiliar GP:
*‘… I don’t even know my doctor’s name. How can I trust someone I don’t even know?’*
Over time, these and other factors have led Saima to mistrust the healthcare system. She is reluctant to engage in remote consultations or have any involvement with the NHS. Previously, Aamir would accompany Saima to her face-to-face appointments and she would have an opportunity to see and talk to nurses and GPs. The shift to remote has made her feel misunderstood, disconnected, and inadequately cared for:
*‘Why should I see some random GP, they don’t know what I need, I know what I need, they think I have some different issue but it is just my BP* [blood pressure] *going high. I can fix it* [with herbal remedies] *, I don’t need treatment.’*

As Saima’s story illustrates, the persona combines age, gender, socioeconomic status, ethnicity/race, digital ability, and other contributors to digital disadvantage, and helps explain how these intersecting influences serve to restrict access or complicate care. Three additional personas are provided in Supplementary Figures S1–S3.

The focus groups found the personas realistic and engaging. Introducing them brought focus to the discussion and encouraged participation. Discussions were often triggered by questions that participants asked about the scenarios encountered by the persona. Patients and patient advocates tended to relate to the persona by sharing their own experiences and stories.

In the sections that follow, we consider some recurring themes from our data that have significant implications for the design (and redesign) of remote and digital solutions.

### Digital services disorient patients

Attempting to access and use digital services often engendered a profound sense of disorientation among participants. Traditionally, patients had traversed a familiar and well-defined path characterised by scheduled appointments and in-person visits to their known primary care provider. They now found themselves grappling uneasily with the intricacies of the multimodal pathways and processes of a digitised service. All patients felt disoriented to some extent in the digital environment, but those at multiple disadvantage (in our sample, older adults, low income, limited English proficiency, unwell in various ways, and experiencing racism and discrimination) felt it especially acutely. Their digital skills and experience were usually limited and they found it difficult to articulate their concerns or symptoms effectively. The additional challenges described in the sections below further contributed to an overwhelming sense of isolation and disorientation, exacerbating the feeling of being ‘lost at sea’ amid the digital expanse.

### Digital services disrupt continuity

Participants in our sample felt troubled seeing unfamiliar clinicians for the first time in the digital environment (see the ‘strange floating face’ in Saima’s example, Box 1). In contemporary digitised services, traditional continuity (in which a single clinician supports the patient holistically through their illness trajectory) is difficult to achieve and rarely found. Instead, there is (at best) ‘distributed continuity’, in which various services and providers contribute elements of care that are recorded on a shared patient record.^35^^,^^36^ But although the electronic record may create a more or less coherent digital facsimile of the patient experience that allows different staff members to perceive continuity, from the patient’s perspective the input of multiple providers is experienced as fragmented care, exacerbating their sense of loss and bewilderment.

### Digital interactions may have weak presence

Although the convenience of video consultations was appealing to some participants, the downside was a sense that their provider was not fully present. They described feeling ‘disconnected’ from their clinician, making information exchange and shared decision making difficult. During video consultations, some patients could not discern if clinicians were looking at them, leading to perceived uninterest. A lack of affirming gestures such as head nodding and smiling also contributed to a weak presence. Some felt they had been abandoned by their providers despite a technically successful remote consultation. Several used the expression ‘better than nothing’ to describe their video encounters, conveying the sense that much of importance (a sense of human connection, rapport, and attention) was missing. Several participants described the video consultation as impersonal, and, like Saima, perceived the clinician as not interested in understanding their concerns.

### Digital encounters do not strengthen relationships (and may weaken them)

Even when participants in our sample had successive video consultations or other remote interactions with the same provider, they rarely developed a sense that they were building a strong and positive therapeutic relationship. In contrast with the face-to-face environment, in which the patient–provider relationship is usually strengthened with each successive encounter, this strengthening did not appear to occur with virtual encounters. Indeed, some participants described a worsening of their relationship over repeated virtual encounters (unless preceded with face-to-face encounters).

### Digital services reduce the patient’s sense of agency

Several participants described profound feelings of disempowerment when interacting in the virtual environment. Saima’s narrative illustrates this (see quote beginning *‘He* [the GP] *didn’t even look at me’* in Box 1). All clinician–patient relationships are characterised by unequal power distribution, since the clinician has greater professional knowledge and holds ultimate control over decisions (for example, whether to prescribe or refer), pace (how quickly the conversation runs), and time (when the consultation will end). This power imbalance appeared to be magnified in the digital environment, sometimes to the extent that the patient who was disadvantaged felt complete absence of agency and only engaged with care passively, if at all. As illustrated by Saima (Box 1), this sense of disempowerment appeared to stem from a number of interacting influences including disorientation and inability to navigate the system, unfamiliarity and disconnection from healthcare providers, limited understanding of the language, the fast-paced nature of consultations, and a perception that the provider was not interested in their thoughts and opinions. Some also felt disempowered when family members, often male, took over during the consultations. Uneasiness with using the technology also contributed, especially if there was inadequate support from family to set it up and ensure it was functioning as the consultation unfolded.

### Digital services increase mistrust of health services and providers

As Saima’s narrative (Box 1) illustrates, the disorienting and fragmented nature of remote interactions, weak sense of presence, loss of personal relationships, and (for many) a sense of disempowerment, along with lack of cultural congruence and (in some cases) perceived racial discrimination, seemed to combine to erode the person’s trust in both individual providers and the system as a whole. In many cases, this fed into an escalating cycle of disengagement with services. Many participants commented that trust was essential for establishing a strong therapeutic relationship and ensuring positive healthcare experiences (both virtually and otherwise). Without it, effective care would not result.

### Suggestions to improve digital services

Participants had many suggestions to improve services in a way that reduced disorientation, improved continuity, increased the sense of presence, strengthened relationships with their provider, reduced feelings of disempowerment, and increased trust.

Although many found the virtual environment inherently alienating, they also felt that its challenges could be at least partially overcome through communication, including the provider stating their name, listening attentively, providing clear explanations, and using interpreters or bilingual health advocates when necessary (partly to help with language but also to restore, at least partially, the patient’s sense of agency). Participants also exhorted providers to humanise the virtual encounter by establishing a sense of connection and empathy through active listening, positive body language (for example, smiling), and genuine recognition of patients’ unique needs and perspectives.

Other suggestions included seeing the same (known) provider as much as possible, ensuring adequate technical connection and support, allowing sufficient time to progress the consultation at the patient’s pace, ensuring that the patient’s concerns are surfaced and addressed, and involving them in discussions and decisions.

## Discussion

### Summary

This qualitative study involving interviews, ethnographic observations, and focus groups highlights how remote and digital services can create significant challenges for older patients with multiple markers of disadvantage. Using an intersectional lens we have shown how limited economic, social, and linguistic resources; social and cultural isolation; perceived racial discrimination; low digital, health, or system literacy; and impairments as a result of physical illness and advanced age combine and reinforce one another as individuals attempt — with varying success — to access and receive care through digital modalities. Four contrasting personas were produced, which captured the variety and complexity of how dimensions of disadvantage intersected and influenced identity and actions. The personas illustrated important themes, including a widespread sense of disorientation (and, hence, inability to navigate the system), fractured continuity, limited presence, weak relationships, loss of agency, and a growing mistrust of services and providers.

Broadly speaking, the more markers of disadvantage an individual had, the more difficulty they faced navigating the ‘digital front door’ and remote consultations. For some, the combined effect of profound disorientation, loss of interpersonal relationships and continuity of care, and loss of trust in particular providers and the system led to a cycle of disengagement. This deterred them from seeking health care or engaging in treatment plans at a time in their lives when such care was greatly needed. Although our study was not designed to examine long-term outcomes, this is likely to result in a compounding of the inverse care law, in which patients most in need of health care are least likely to receive it.

### Strengths and limitations

The main strength of this study is the use of an intersectional lens, underpinned by theory, to produce personas based on a detailed exploration of the lived experiences of a sample of older patients with multiple markers of disadvantage. A multilingual researcher working closely with community-based provider organisations was able to access a group of service users at the margins, whose ‘subjugated knowledges’ have historically been devalued, misinterpreted, excluded, or trivialised, and who have rarely been represented in research.^37^

The main limitation is that the sample was relatively narrow and homogeneous: first-generation South Asian immigrants to the UK who were older adults. Although our method for generating personas is likely to be generalisable to other disadvantaged and marginalised groups, the specific findings are not. A limitation thus far is that the personas have not been tested in practice but this phase is planned. An additional limitation is that the focus groups were conducted in English, which excluded most of the study participants.

### Comparison with existing literature

Our findings align with wider literature on what is important in clinician– patient interactions in primary care generally, including a system that is accessible and navigable,^38^ continuity of care,^35^^,^^36^^,^^39^ therapeutic presence, a positive clinician–patient relationship,^40^ patient-centredness,^41^ patient empowerment,^42^ and the crucial importance of trust in maintaining engagement with health services.^43^ Our contribution is novel in demonstrating how these closely interrelated issues play out differently in the context of patients who are multiply disadvantaged and are attempting to access digital services.

We also make an important contribution to the literature on user personas. Personas have been used previously to enhance understanding of, and help design, services for older people with complex needs.^29^^,^^30^ Of note is the Esther model in Sweden, developed by a team of physicians, nurses, and other providers with the goal of improving patient flow and coordination of care across a six-municipality region.^32^ Like our study, the Esther model emphasises the power of patients’ stories, which are elicited and collected to illustrate how patients’ lives are affected by their health challenges and their experiences in getting care. ‘Esther’ is not a real patient, but her persona as an ailing but competent older woman with a chronic condition and occasional acute exacerbations inspired a region-wide programme of service improvement, including an ‘Esther network’ of provider organisations and ‘Esther coaches’ who worked with them to achieve significant reductions in hospital admissions, length of stay, waiting times, and readmissions.^32^

Our contribution to the user persona literature is novel because, unlike Esther (who is old but living independently, fluent in Swedish, engaged with services, and not obviously ‘at sea’ in the system), the personas developed in this study specifically represent older people with multiple markers of disadvantage who are, to a greater or lesser extent, ‘at sea’ and disengaged with the system. Our contribution also focuses specifically on remote and digital services.

### Implications for practice

Our goal in undertaking this study was to explore whether user personas reflecting multiple disadvantage could be developed and whether these would prove useful when designing or redesigning digital primary care services. The suggestions by our participants (see last section of Results: Suggestions to improve digital services) provide some general guidance.

We envisage that next steps include using the personas to prompt creative discussions about an actual service, asking questions such as ‘How can we make this service more accessible and empowering for people like Saima?’ The personas could also inspire the development of novel measures of system success, reflecting the issues highlighted as important to the users who are most disadvantaged. But such efforts must acknowledge the reality of system pressures. Measures that will optimally support and empower Saima will be costly and time consuming, and hence have an opportunity cost. In short, additional resources are needed for practices serving deprived populations.

Two surprising and troubling findings from our study have potential implications for policy. First, the challenges of digital services (disorientation, fragmentation, and a sense that digital encounters are often impersonal and transactional) tend to produce a profound sense of disempowerment, loss of trust, and disengagement with the system in patients who are disadvantaged. Second, digital interactions may not strengthen clinical relationships but may actually worsen them when patients are multiply disadvantaged, leading in some cases to a breakdown of trust. If replicated in other studies, these preliminary findings suggest that a ‘digital first’ policy could actually be harmful to the most vulnerable groups, and it is possible that the human and economic costs of pushing the digital model in these groups could be prohibitive.

Within the current healthcare landscape, prevailing incentives tend to prioritise urgent access and initial contact assessments, which may result in surface-level interactions and limited support for individuals experiencing intersectional disadvantage. The current system inadvertently diverts the attention, effort, and resources of practitioners away from the ongoing, relational aspects of primary care. If Saima has lost trust and becomes disengaged from the system, she may fail to seek care when acutely unwell (or, she may go directly, and inappropriately, to the emergency department), and control of her long-term conditions will deteriorate, increasing the risk (and cost) of acute complications such as stroke. For these reasons, measures promoting relationship-based care and maintaining patient engagement in patients who are multiply disadvantaged, although costly in the short term, are likely to pay off in the medium and long term.

One potential approach to supporting patients who are multiply disadvantaged involves the implementation of coding systems, alerts, or other reasonable adjustments to identify them, helping to ensure that staff are aware and oriented to addressing their specific needs. Such an approach would likely need to be combined with support for bespoke solutions, since, as intersectionality theory stresses, disadvantage plays out differently in everyone and there is no one-size-fits-all solution.

Another approach strongly supported by our data is to prioritise traditional continuity of care with a single clinician (or small buddy group or micro-team)^44^ for patients who are multiply disadvantaged. In-person appointments could be offered routinely as an initial step to establish a therapeutic relationship, build trust, and surface the patient’s priorities and concerns ahead of follow-up consultations by phone or video.

It is also important to recognise and mitigate technological disparities among patients. Technical support, user-friendly interfaces, and availability of multilingual resources can substantially enhance patients’ ability to independently and confidently navigate digital healthcare platforms. We applaud the provision of digital training among marginalised populations — for example, through libraries and faith organisations.^45^^–^47 Another model that might help those with weak digital skills (and that could help retain and strengthen trust) is a locality-based digital hub, based (for example) in community centres and staffed by healthcare assistants who support the patient both technically and culturally to have a digital consultation. Such hubs have been successful in remote parts of Scotland to overcome geographical distance;^48^ the model might be adapted in inner-city populations to overcome cultural distance.
